# Self-Inflicted Traumatic Bilateral Upper Extremity Amputation as a Suicidal Gesture?

**DOI:** 10.7759/cureus.17176

**Published:** 2021-08-14

**Authors:** Chinwe Ogedegbe, Ndausung Udongwo, Ayoola Kalejaiye, Abbas Alshami, Nasam Alfraji, Magdalene Spariousu

**Affiliations:** 1 Emergency Medicine, Hackensack University Medical Center, Hackensack, USA; 2 Internal Medicine, Jersey Shore University Medical Center, Neptune City, USA; 3 Internal Medicine, Montefiore Medical Center, Moses Campus, Bronx, USA; 4 Psychiatry, Hackensack University Medical Center, Hackensack, USA

**Keywords:** traumatic amputation, upper extremity amputation, depression, pain, female gender, suicide attempt

## Abstract

Traumatic amputations are partial or complete dismemberment of part of the human body (usually one limb) due to an injury that involves a component of blunt force trauma. It is usually caused by accidental events and only very rarely due to suicidal events. A 37-year-old female with major depressive disorder attempted suicide by placing her forearms on a railroad track, resulting in traumatic bilateral upper extremity amputations. Emergency Department resuscitation was initiated as the patient was taken immediately to the operating room; however, restoration of the limbs was unfeasible, and the patient had successful debriding and fashioning a flap to the distal ends of the upper extremities after hemostasis was achieved. Depression may still be an undertreated clinical entity in our society, and many preventable causes of suicide are attempted each year. Evidence exists that suggests severe suicide attempts occur generally in men and minor suicide attempts, or so-called suicidal gestures, occur generally in women. This case questions this notion.

## Introduction

A traumatic amputation is the partial or complete dismemberment of part of the human body; frequently, but not limited to, one of the four extremities, and is the result of an accident or injury commonly involving a component of blunt force trauma [1]. Traumatic amputations are often fatal requiring immediate medical intervention, and intravascular volume resuscitation to prevent bleeding and hemorrhagic shock. Patients that are status-post traumatic amputations undergo extreme physio-compensatory changes to maintain mean arterial pressure, as well as psychological trauma. Over time, they experience unbearable neuropathic pain from the injured nerves, and their functional status decreases [1]. The prevalence of clinically relevant depression is about 45% in orthopedic trauma patients, while post-traumatic stress disorder (PTSD) frequently occurs in upper extremity amputees [2,3]. The purpose of this case report is to highlight a unique case, in which a 37-year-old female, suffering from major depressive disorder (MDD), attempted suicide by placing her forearms on a railroad track, resulting in traumatic bilateral upper extremity amputations.

## Case presentation

A 37-year-old Caucasian female, with a clinical history of dysthymia and MDD, presented to the emergency department status-post suicide attempt by lying down prone, perpendicular to a railroad track. Both arms were supinated and rested upon the railroad tracks at the mid-forearm. In the field, it was reported that the train conductor witnessed the patient lying down with arms exposed and attempted to emergently stop the train. His attempts were futile, as the train traveling at a speed of approximately 35 mph resulted in the amputation of both upper extremities (below the elbow). Emergency medical services were notified immediately. The paramedics were successful in controlling the bilateral hemorrhage by applying tourniquets. After assessing no other obvious injuries, bulky sterile dressings were applied to the ends of the bones. The patient was then transported to the emergency department. Replantation of the limbs was not possible due to massive crushing and degloving of the amputated forearms.

**Figure 1:**
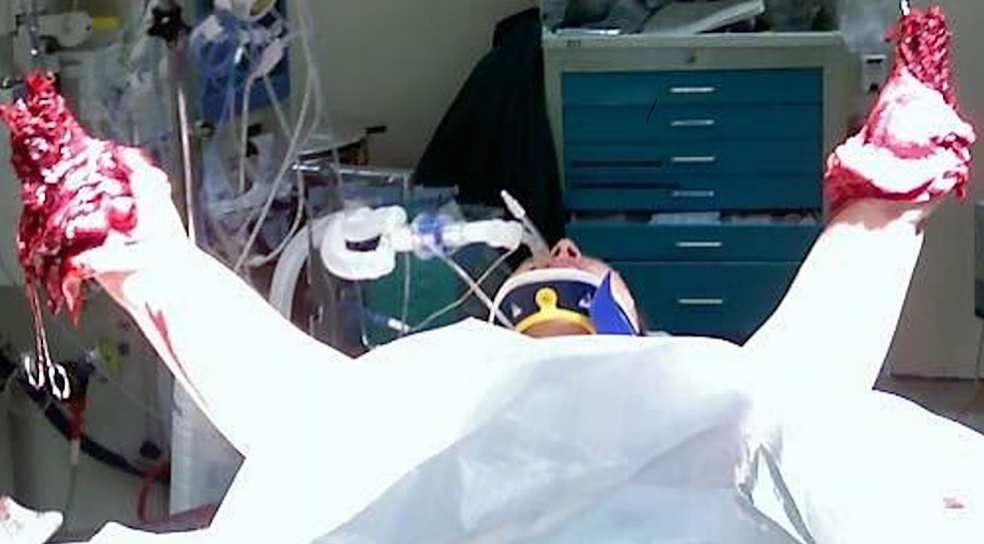
The status of our patient during the operation.

X-ray imaging of the upper extremities demonstrated intact humerus bilaterally. Comminuted fractures were found on the remnants of the radius and ulna bones. Furthermore, no foreign bodies were visualized. The patient was immediately taken to the operating room (Figure 1).

The orthopedic and plastic surgeons consulted, jointly worked on the patient in the operating room. The elbow joints were observed to be intact and in proper anatomical alignment. Each arm was debrided at the stump until a determined viable tissue plane was found. Copious amounts of irrigation (nine liters per extremity) via pulse lavage were done. A soft tissue viable flap was then folded over to cover the exposed wound and stumps, after myofascial reconstruction/repair.

During the post-operative interview, the patient was not very cooperative and gave minimal information. However, the psychiatric consultants were able to extract some information from the patient. Apparently, she had severe depression with no prior suicide attempts. The patient then disclosed to the medical team that she felt this was a fool-proof means of lacerating her wrists. Furthermore, it was concluded that there were no inciting events that prompted her to attempt suicide. The patient refused management of her depression but was eventually transferred to the inpatient psychiatry unit due to the recent suicide attempt. Blood alcohol content and toxicology screen were both negative.

## Discussion

In the US, approximately 1.7 million people are living with the loss of a limb [4]. Approximately 135,000 people in the US undergo amputations per year, with African American males having the highest incidence [5]. The prevalence of traumatic amputations from 1988 to 1996 was 68.6 per 100,000, with upper extremities accounting for the majority of incidents [6]. Furthermore, traumatic amputations of the lower extremities were 31 per 100,000 [6]. The rates of traumatic amputations have declined by approximately half over the past 20 years. Industrial crush injuries of the dominant right hand in young adult males comprise the majority of below-elbow traumatic amputations in the US [7]. Motor vehicles, trains, close-range shotguns, power saws, and natural disasters are among the most common causes as well [7].

A review of the medical literature has shown that up to two-thirds of patients suffering from depression are not aware that there is treatment [8]. Of those diagnosed with depression, approximately 50% receive treatment, of which 20% receive appropriate treatment as per the American Psychiatric Association (APA) [8,9]. Also, approximately 6%-12% of the US population at some point in their lives have an episode of major depression, with suicide accounting for 42,000 deaths yearly in the US, being the eight-leading cause of death [10]. Women have been reported to have a higher number of suicide attempts when compared to men, with the latter reported to have more successful attempts [11].

An underlying myofascial repair was indicated in this case. Equi-distance anterior and posterior flaps allowed for the closure of the wound on the right. However, an unequal flap design on the left was done. When amputation is secondary to trauma, this poses a great challenge to the trauma team. Often, the level of amputation is unclear, and the patient may present with a significant avulsion and/or crush component. In addition, traumatic amputations expose much more eviscerated tissue to the external environment, putting the patient at a significantly higher risk of superimposed infection.

The current recommendation for traumatic amputations, with a high index of suspicion for contamination, calls for primary debridement and delayed closure of the stump, especially in grossly contaminated wounds [12]. This decreases the likelihood that contaminants not apparent on initial examination may be included within the wound and allows the wound to present its viability prior to closure. In patients with proximal below-elbow amputations, as in the patient presented here, forearm rotation is rarely preserved. In general, the more distal the below-elbow amputation is, the better the amputee will function [7]. Below-elbow amputation also offers patients the option to use both arms to grasp objects and function properly, a feat that is not possible with bilateral above-elbow amputations.

When feelings of depression or suicidal ideation become apparent to health care professionals, it is important to act promptly. Feelings of hopefulness, withdrawal, and suicidal ideation may point to an impending attempt. Having patients seek help or admitting the patient involuntarily is recommended and can go a long way in preventing catastrophic outcomes.

This is an original case report of particular interest to orthopedic-trauma and psychiatry. We were unable to find in the literature any other occurrences of using a train to lacerate one’s wrists in a suicide attempt.

## Conclusions

We have presented an atypical case of traumatic amputation in a 37-year-old Caucasian female suffering from major depressive disorder, who attempted suicide by placing her forearms on a railroad track, resulting in traumatic bilateral upper extremity amputations. Clinicians should be more vigilant to women with major depression; this case makes us aware that women could also have severe suicide attempts.
